# Glucan from* Oudemansiella raphanipes* suppresses breast cancer proliferation and metastasis by regulating macrophage polarization and the WNT/β-catenin signaling pathway

**DOI:** 10.7150/jca.89873

**Published:** 2024-01-01

**Authors:** Gulimiran Alitongbieke, Xiuru Zhang, Fukai Zhu, Qici Wu, Zhichao Lin, Xiumin Li, Yu Xue, Xuebin Lai, Jiexin Feng, Rongjie Huang, Yutian Pan

**Affiliations:** 1 Engineering Technological Center of Mushroom Industry, Minnan Normal University, Zhangzhou, Fujian 363000, People's Republic of China.; 2 Department of Breast Surgery, Zhangzhou Affiliated Hospital of Fujian Medical University, Zhangzhou, Fujian 363099, People's Republic of China.; 3 Department of General Surgery, Zhangzhou Affiliated Hospital of Fujian Medical University, Zhangzhou, Fujian 363099, People's Republic of China.

**Keywords:** *Orp*, breast cancer, macrophage polarization, WNT/β-catenin signaling pathway, inflammation

## Abstract

**Background:** The glucan extract of *Oudemansiella raphanipes* (*Orp*) has multiple biological properties, similar to extracts of other natural edible fungi. Drugs traditionally used in cancer treatment are associated with several drawbacks, such as side effects, induction of resistance, and poor prognosis, and many recent studies have focused on polysaccharides extracted from natural sources as alternatives. Our study focuses on the therapeutic role and molecular mechanism of action of *Orp* in breast cancer progression.

**Methods:** MMTV-PyMT transgenic mice were used as the spontaneous breast cancer mice model. Immunoblotting, hematoxylin-eosin staining, immunohistochemistry, and immunofluorescence were used to evaluate the tumor behaviors in breast cancer. The inflammatory cell model was constructed using TNF-α. Macrophage activation and WNT/β-catenin signaling were assayed using western blotting and immunofluorescence.

**Results:*** Orp* management significantly inhibited tumor growth and promoted tumor cell apoptosis in MMTV-PyMT transgenic mice. Besides, the* Orp* challenge also attenuated the ability of breast tumors to metastasize into lung tissues. Mechanistically,* Orp* treatment restrained the polarization of M1 macrophages to M2 macrophages and suppressed WNT/β-catenin signaling in mouse tumor tissues, which implied that* Orp*-mediated tumor inhibition partly occurred *via* regulating the inflammatory response. Findings from *in vitro* experiments confirmed that* Orp* inhibited the TNF-α-induced nuclear transportation of β-catenin, thus preventing inflammation signaling and the expression of c-Myc in MCF-7 cells.

**Conclusion:*** Orp* inhibits breast cancer growth and metastasis by regulating macrophage polarization and the WNT/β-catenin signaling axis. The findings of this study suggest that* Orp* may be a promising therapeutic strategy for breast cancer.

## Introduction

Breast cancer was the most commonly diagnosed cancer (24.2% of total cancer cases) and the leading cause of cancer-related deaths (15% of total cancer deaths) among females worldwide 1. The increasing incidence of breast cancer, severe side effects of traditional cancer therapies (chemotherapy and radiation therapy), emergence of acquired resistance, and poor prognosis suggest an urgent need for effective anticancer drugs with high safety to overcome the above clinical problems 2. The extracts of natural products are gaining increasing popularity as promising therapeutic strategies for their potential to fight cancer 3. Particularly, polysaccharides extracted from natural sources (mushrooms, plants, algae, etc.) have been studied extensively and confirmed to exhibit strong antitumor and immunoregulatory properties, with minimal side effects 4, 5. Therefore, the development of anticancer polysaccharides from natural resources can contribute to breast cancer therapeutics.

* Oudemansiella raphanipes* (*Orp*), known as “Heipijizong” or “Black Termite Mushroom,” is a commercial mushroom with high nutritional value and excellent and unique flavors and has been widely cultivated in China in the past few years 6. *Orp* polysaccharides can effectively scavenge 2,2'-azino-bis(3-ethylbenzothiazoline-6-sulfonicacid) and 1,1-diphenyl-2-picrylhydrazyl radicals and exhibits antioxidant properties 7. However, its antitumor properties have rarely been reported in the past few years. The natural antioxidants present in this extract do not have noticeable side effects and can serve as effective treatment agents by counteracting the oxidative stress caused by excess reactive oxygen species 8. Polysaccharides derived from mushrooms with medicinal properties are attractive treatment agents for managing oxidative stress and as functional foods for humans. Accumulating evidence indicates that the edible fungus-derived glucan can regulate tumor-associated macrophages (TAMs) in Lewis lung cancer 9 and can inhibit tumor growth and lung metastasis by repressing tumor-induced angiogenesis 10. Findings from studies have shown that β-glucan from *Lentinus edodes* (LNT) suppresses cell proliferation and promotes apoptosis in estrogen receptor (ER)-positive breast cancers. Other studies have shown that LNT inhibits tumor growth by suppressing cell proliferation and enhancing apoptosis *via* the PI3K/AKT/mTOR, NFκB, ERK, Erα, caspase, and p53-dependent pathways 11-13. Findings from previous studies have shown that the novel neutral polysaccharide (lgps-1) isolated from *Panus giganteus* can block cell proliferation and induce apoptosis in liver cancer cells 14. Additionally, polysaccharides (named MHP-1) from *Mortierella hepialid* were shown to inhibit breast cancer metastasis, lower the expression of TGFβ type I receptor kinase (ALK5) and vimentin, and enhance the expression of E-cadherin 15. However, few reports have discussed the effects of *Orp* polysaccharides on breast tumors.

One of the hallmarks of malignancy is the polarization of tumor-associated macrophage (TAMs) from a pro-immune (M1-like) phenotype to an immune-suppressive (M2-like) phenotype 16. TAMs act as the key determinant in regulating breast cancer metastasis and progression. Targeting TAMs is a novel strategy against breast cancer 17, 18. In a murine breast cancer model, Oghumu et al 19 confirmed that CXCR3 deficiency enhances tumor progression by promoting macrophage M2 polarization as well as that cyclooxygenase-2 (COX-2) inhibition blocks M2 macrophage differentiation and suppresses breast cancer metastasis. Findings from a large number of studies have shown that inducible nitric oxide synthase (iNOS) is primarily produced by macrophages, and the inflammatory response it mediates is associated with AS and MI/RI pathology 20. WNT/β-catenin signaling is involved in the cross-talk between cancer cells and TAMs 21. M2 macrophage polarization was found to promote WNT/β-catenin signaling in cholangiocarcinoma cells 22. It is a potential treatment for lung cancer if TAMs can be reprogrammed by targeting WNT/β-catenin/FOSL2/ARID5A signaling 23. Additionally, enhanced WNT/β-catenin signaling can promote epithelial-mesenchymal transition (EMT) in breast cancer cells 24. The suppression of miR-454-3p-mediated Wnt/β-catenin signaling antagonists promotes breast cancer metastasis 25. Since polysaccharides extracted from *Orp* presumably possess strong immunomodulating effects, we speculate that the anti-tumor effect of Orp could be implicated in the regulation of macrophage polarization and WNT/β-catenin signaling.

To demonstrate the hypothesis, the spontaneous breast cancer mice model was used to explore the role of* Orp* in breast cancer growth, proliferation, apoptosis, and lung metastasis. Additionally, we investigated whether macrophage polarization and the WNT/β-catenin signaling pathway influence the progression of breast cancer under the regulatory effects of* Orp*. Our data shed light on the mechanism underlying the anti-tumor effects of *Orp*.

## Materials and methods

### Preparation of Orp

*Orp* was purchased from the Mengdel (Xiamen) Biotechnology Co., LTD, as a commercial product cultivated in Fujian Province of China. The process of polysaccharide extraction was adopted from previously reported methods 26. β-glucan was extracted from* Orp* in our laboratory. Briefly, the dried sporophores of* Orp* were crushed and subjected to extraction with water (w/w = 1:40) at 100 °C for 2 h. The residues obtained after centrifugation were subjected to extraction using 2% NaOH (w/w = 1:3) for 2 h. After washing with water for neutralization, the residues were filtered, dried, and crushed. Following this, the residues were subjected to extraction using water (w/w = 1:40) at 100 °C for 2 h, centrifuged, concentrated, and dried, following which *Orp* was isolated.

### Purity analysis

A small quantity of *Orp* was weighed and used to prepare a 2 mg/mL solution. The solution was filtered at 0.45 μm. High Performance Liquid Chromatography (HPLC) separation was performed on Waters sugar pak-1 columns (7.8 mm × 300 mm), and the samples were subjected to detection using a refractive index detector. The mobile phase was ultrapure water. The column temperature was maintained at 85 °C. The flow rate was set at 0.6 mL/min, and the pressure was 18.4 bar.

### Cell culture and cell treatment

Human breast cancer MCF-7 cells were obtained from the Type Culture Collection of the Chinese Academy of Sciences (Shanghai, China). The cells were cultured in high-glucose Dulbecco's modified eagle medium (DMEM) (Gibco, USA) supplemented with 1% penicillin/streptomycin and 10% (v/v) (Gibco) fetal bovine serum (FBS) (Gibco, USA) and maintained at 37 °C in a 5% CO_2_ incubator (Thermo, USA). Cells were seeded in 6-well plates or 24-well plates at at a density of 10^5^ cells/cm^2^ the day before the experiments. When 70%-80% confluence was achieved, *Orp* (125, 250, 500, 1000, and 2000 μg/mL) were added to the plates for 24 h incubation and 20 ng/mL TNF-α were added into cell culture 30 min before the end of Orp treatment. For the treatment of inhibitor, 20 μM MG132 (Selleck, USA) was used to block the ubiquitin-proteasome pathway 6 h before the treatment of TNF-α and Orp. Following this, cytosolic protein and nuclear protein were extracted using a nuclear/cytoplasmic protein separation kit.

### Animals and treatment

Specific-pathogen-free (SPF) mouse mammary tumor virus (*MMTV*) - Polyoma Virus middle T antigen (*PyMT*)/MMTV-PyMT transgenic mice (background: FVB), which are well recognized as a breast cancer model, were obtained from the School of Pharmaceutical Sciences, Xiamen University. All animals were fed normal mouse food and water ad libitum under SPF conditions at a fixed temperature of 20 ± 2 °C and a 12 h dark-light cycle under a relative humidity of 60%-70%. All procedures involving animals were conducted in accordance with and after approval from the Animal Ethics Committee of Minnan Normal University (ethical approval number: IACUC-IME-2021-010). Eight-week-old female PyMT transgenic mice with similar initial body weight and growth status were selected and divided into two groups randomly: control and *Orp*-treated group (5 mice/group). The mice were challenged by an intravenous injection of 1 mg/kg *Orp* (per mouse) for 7 weeks in the *Orp*-treated group. Mice in the control group were challenged with normal saline for dissolving the drug. Tumor onset was recorded as the initial injection day. Tumor growth in all of the ten mammary glands in each mouse was evaluated using biweekly palpations and measured. At the end of the treatment, the mice were executed with cervical dislocation, and the breast tumor tissues and lung tissues were immediately dissected, photographed, and weighed. Subsequently, a part of the tumor tissue and lung tissue were used for protein extraction, and another part was used for immunohistochemistry and immunofluorescence staining.

### HE staining

Mouse tumor tissue and lung tissue samples were cut and immersed in 4% paraformaldehyde at 4 °C for more than 24 h. Following this, the samples were dehydrated with graded ethanol, immersed in xylene, and embedded in paraffin. The embedded tissues were cut into 4 μm sections. Following dewaxing, the sections were stained with HE (Beyotime, China). Each group of samples was observed using an optical microscope (Olympus IX71, Japan).

### Immunohistochemical analysis

Briefly, the sections were retrieved in citric acid buffer (pH 6.0) by microwave antigen retrieval and blocked with 0.3% hydrogen peroxide for terminating endogenous peroxidase activity at room temperature for 20 min. Following this, nonspecific protein binding was blocked by incubation with 2% BSA. This was followed by overnight incubation with the primary antibody against proliferating cell nuclear antigen (PCNA) (AF0239, Affinity; diluted 1:100) or MMP-9 polyclonal antibody (AF5228, Affinity; diluted 1:100) at 4 °C. Next day, the proteins were treated with the secondary antibody, conjugated with horseradish peroxidase, and developed according to the manufacturer's instructions (IHC staining module, Beijing Zhongshan Biotechnology, China). The sections were counterstained using hematoxylin and immunohistochemical staining, and the results were observed using a light microscope (Olympus IX71, Japan).

### Immunofluorescence staining

Four-micrometer-thick paraffin sections were subjected to immunofluorescence staining overnight with the primary antibodies against CD86 (sc-19617, diluted 1:100) or monoclonal CD206 (14-9760-80, Invitrogen, diluted 1:100) at 4 °C. On the second day, the slides were washed with 1× PBS for 5 min each time and incubated with the corresponding secondary antibody (Molecular Probes, USA, diluted 1:200) for 1 h at room temperature. Dihydrochloride (DAPI, Beyotime, China, diluted 1:300) was used to visualize the nucleus. CD86 and CD206 were detected under a fluorescence microscope (DIM8, Laica, Germany). The cells that yielded positive staining results were counted using the Image Pro Plus software.

### TUNEL assay

The apoptosis of tumor cells in the control and the *Orp*-treated group was detected using a one-step TUNEL apoptosis assay kit (Beyotime, China). Briefly, PBS (Beyotime, China) supplemented with 0.5% Triton X-100 (Beyotime, China) was added to the tissue slices and incubated for 5 min at room temperature. Subsequently, 50 μL of the TUNEL test solution was added to the slides, and the slides were incubated at 37 °C for 1 h in the dark. Finally, DAPI staining was used to visualize the nucleus. After blocking with an anti-fluorescence quenching mount, the apoptotic cells were observed under a fluorescence microscope (Leica DIM8, Germany).

### Western blotting

The tumor tissue was cut into small pieces and dissolved on ice for 20 min by adding RIPA lysate (Beyotime, China). After centrifugation at 12,000 rpm for 5 min, the supernatant was collected as the tumor tissue protein extract. For the extraction of cytosolic and nuclear proteins, MCF-7 cells were lysed using a nuclear/cytoplasmic protein separation kit. Following this, proteins from tumor/lung tissues, cytoplasm, and nuclei of MCF-7 cells were stored at -80 °C for western blot analysis. Equal quantities of protein were subjected to sodium lauryl sulfate-polyacrylamide gel electrophoresis (SDS-PAGE). The proteins were then transferred to a PVDF membrane (Beyotime, China) and blocked using a 5% skim milk powder (Beyotime, China) for 1 h. Following this, the proteins were treated overnight at 4 °C with the following primary antibodies at the appropriate dilutions: PARP (DF7198, Affinity), PCNA (AF0239, Affinity), MMP-9 (AF5228, Affinity), N-cadherin (T55015, Abmart), E-cadherin (AF0131, Affinity), P-IKK (TP56290, Abmart), P-IκB (TP70389, Abmart), ARG1(T55101, Abmart), iNOS (AF0199, Affinity), WNT1 (AF5315, Affinity), FZD2 (AF5282, Affinity), β-catenin (AF6266, Affinity), p-β-catenin (DF2989, Affinity), c-Myc (AF0358, Affinity), cyclin-D1 (AF0931, Affinity), GSK3β (BF8003, Affinity), P-GSK3β (AF2016, Affinity), HSP60 (AF0184, Affinity), β-actin (T0022, Affinity). After washing the membrane, the corresponding secondary antibody (Beyotime, China) was added for incubation for 1 h. Chemiluminescence detection was conducted using an enhanced chemiluminescence reagent (Beyotime, China). After development and fixing treatment, the film was photographed using a gel imaging analysis system (UniCel DxI800, Beckman Coulter, USA).

### Statistical analysis

HE and IF staining results were analyzed using Image J. The quantitative analysis of blots was performed using Quantity One software. Data were statistically analyzed using a one-tailed Student's *t*-test or one-way ANOVA using GraphPad Prism 8.0 software. *p < 0.05 and **p < 0.01 were used to indicate a significant difference and a considerably significant difference, respectively.

## Results

### Structural characterization of Orp and role of Orp in breast cancer growth in PyMT transgenic mice

**Figure 1:**
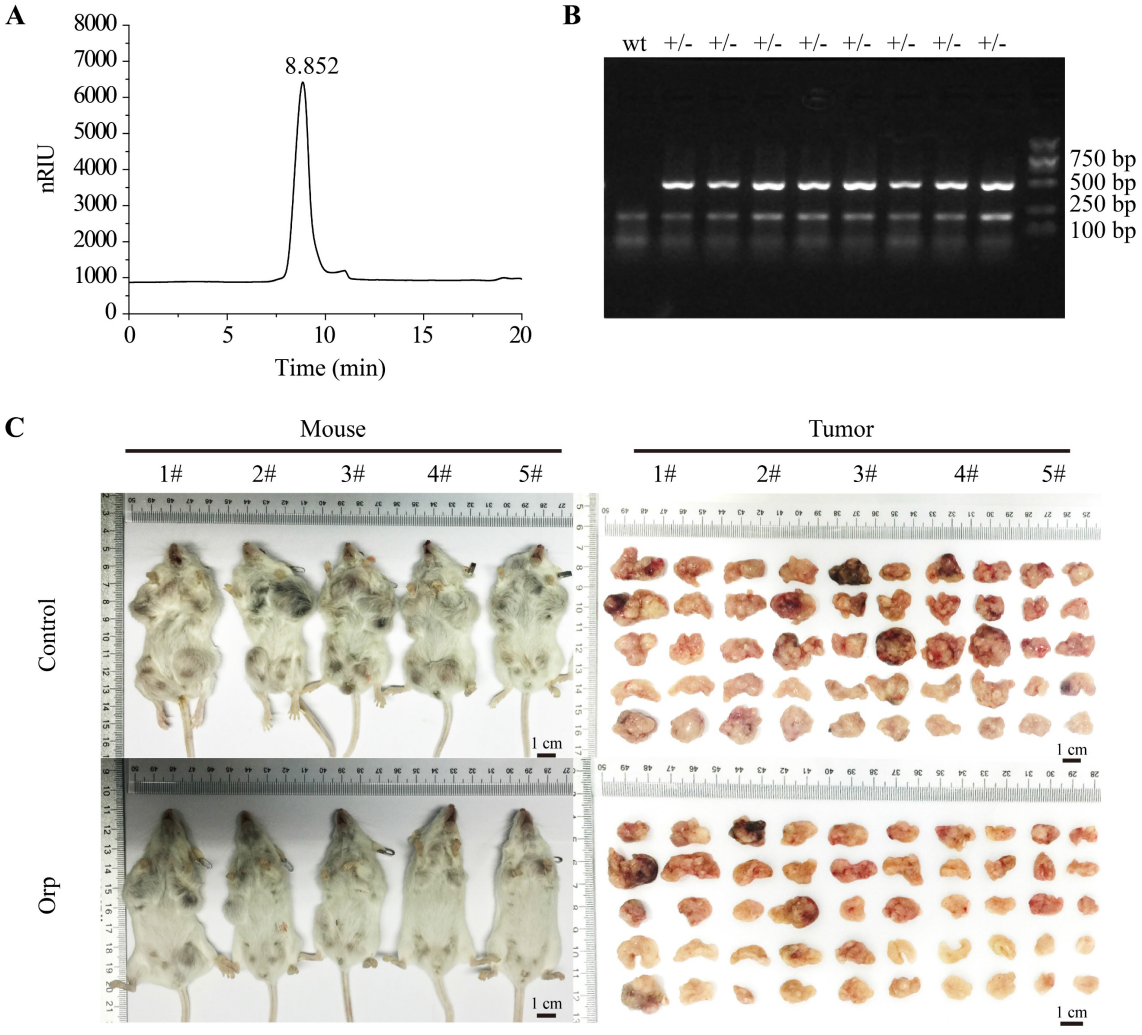
** Effects of *Oudemansiella raphanipes* (*Orp*) on tumor growth in PyMT transgenic breast cancer mice. (A)** High‑performance liquid chromatography analysis of Orp‑derived glucan. (inset) Photographs of Orp dispersed in PBS. **(B)** Identification of MMTV-PyMT mice genotype (lane +/-: MMTV-PyMT; wt: non-MMTV-PyMT). The primer sequence was listed as below, forward: 5'-GGAAGCAAGTACTTCACAAGGG-3'; Reverse: 5'-GGAAAGTCACTAGGAGCAGGG-3'. **(C)** Images of tumor-bearing mice and mammary tumor size observation in the *Orp*-treated group and the control group in PyMT transgenic mice (n=5 each group).

As shown in Figure 1, there was no other peak appeared during the retention time of *Orp* (Figure 1A), which suggested that single β-glucan was isolated sufficiently to perform further experiments for investigating the preventive effect of Orp (β-glucan from* Oudemansiella raphanipes*) on breast cancer cells. Eight-week-old female MMTV-PyMT transgenic mice with spontaneous breast cancer were selected randomly as experimental therapeutic experimental objects. The genotypic identification of MMTV-PyMT transgenic mice was presented in Figure 1B. In the present study, PyMT transgenic mice showed significant breast tumor contour characteristics when they were 3 months old (12 weeks). When treated with *Orp* for 6 weeks, the sizes of all ten mammary gland tumors in each mouse were significantly inhibited compared to those in the control group (Figure 1C). Additionally, the tumor weight in each pair of mammary glands was notably lesser in the *Orp* group than in the control group (Figure S1A). In contrast, the body weight and weights of the heart, liver, spleen, lungs, and kidneys remained unchanged in both groups (Figure S1B, 1C). This result indicated that *Orp* treatment inhibited breast tumor growth of PyMT transgenic mice and had minimal side effects.

### Orp management regulates tumor growth and the apoptosis of breast tumors

To explore the reason for the *Orp*-mediated anti-tumor effect, we monitored the proliferation- and apoptosis-related events in the tumor tissues. HE staining results revealed that the mammary tissues and glands were filled with tumor cells in the control group (Figure S2A, the left row). Additionally, *Orp* treatment reduced the expression of PCNA compared to that in control mice (Figure S2A, the middle row). Apoptosis was also confirmed in the TUNEL staining assay. The results showed that *Orp* challenge observably enhanced the apoptotic signals in tumor tissues compared with that in control tumors (Figure S2A the right row). Based on the results of the immunoblotting experiments, treatment with *Orp* not only reduced the expression of full-length PARP (implying that *Orp* promoted the cleavage of the apoptotic protein PARP) in tumor tissues but also inhibited the protein expression of PCNA (Figure S2B). Therefore, *Orp* significantly inhibited tumor cell proliferation and promoted tumor cell apoptosis, thereby inhibiting breast cancer progression.

### Orp affects lung metastasis in PyMT transgenic breast cancer mice

**Figure 2:**
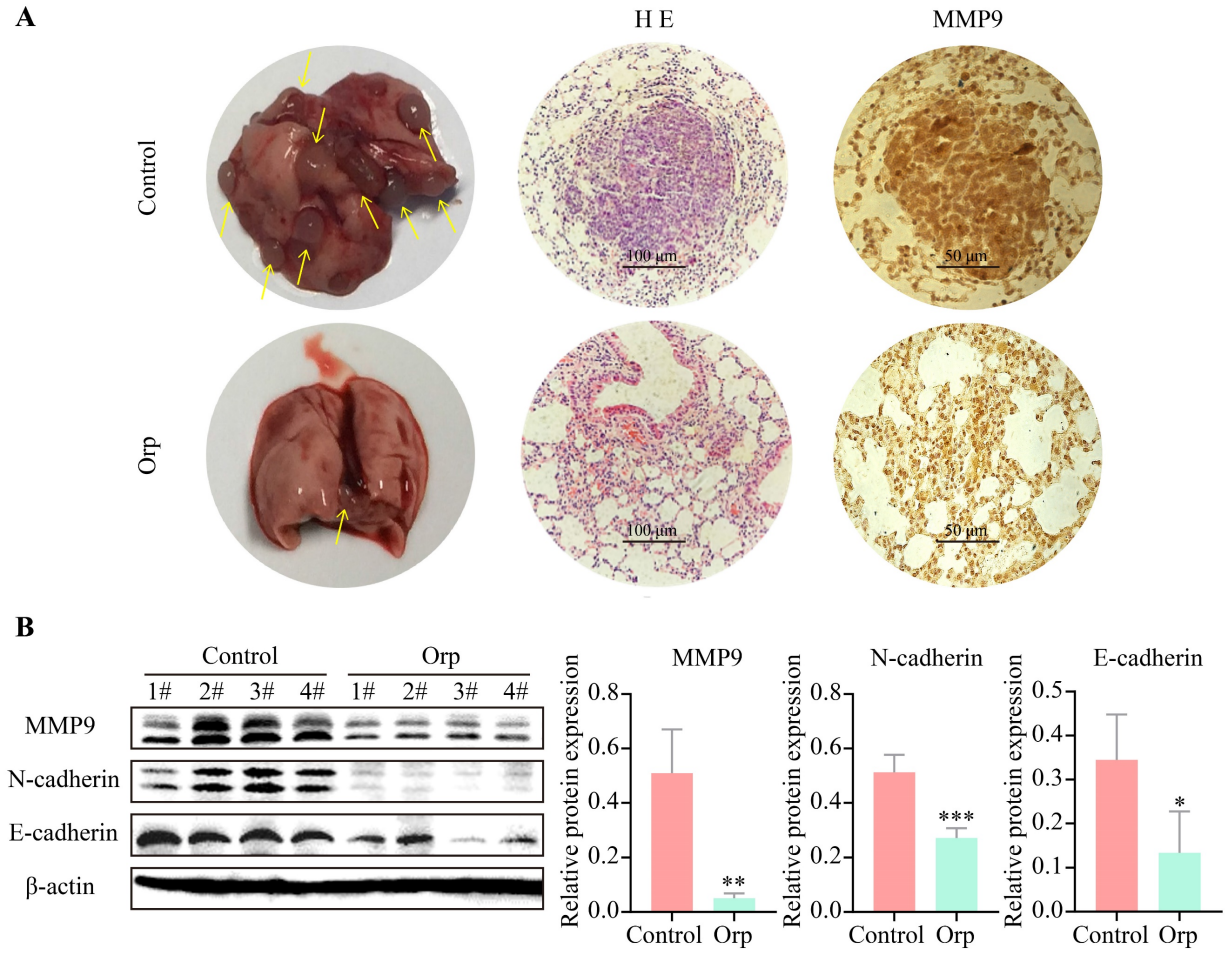
** Effects of *Orp* on the lung metastasis of breast tumor. (A)** Images of lung tissues, HE staining, and immunostaining of MMP-9 in the lung tissues of the Orp-treated group and control group (Scale bar: 100 μm and 50 μm).** (B)** The expression and semi-quantitative statistics of MMP9, N-cadherin, and E-cadherin proteins in different groups, as analyzed by western blotting and photographed using ImageJ. Data are presented as means ± SD, n≥3. **P<0.01; ***P<0.001.

Lung metastasis from breast cancer is a common phenomenon in PyMT transgenic mice. Herein, the role of *Orp* in the lung metastasis of breast cancer cells was also determined. Mice were dissected to count the number of lung tumor metastases (Figure 2A, the left row). The results revealed more tumor foci in the control group. In contrast, *Orp* significantly inhibited pulmonary metastasis. HE staining of lung tissues indicated the presence of multiple tumor foci, inflammatory lesions, severe fibrosis, and the aggregation of a large number of inflammatory cells in the lung tissues of the control group (Figure 2A, the middle row). However, in the *Orp* group, the tissues showed fewer tumor foci, lesser inflammatory cell infiltration, and fewer inflammatory lesions. The subsequent MMP9 immunohistochemical staining of lung tissues indicated that the number of MMP9-positive cells in the *Orp* group was significantly less than that in the control group (Figure 2A, the right row). Additionally, western blotting results showed that *Orp* significantly inhibited the expression of matrix metalloproteinase MMP9 (Figure 2B, band 1). Besides, *Orp* treatment decreased the levels of N-cadherin and E-cadherin in lung tissues compared to that in the control group (Figure 2B, bands 2 and 3). The above results demonstrate that *Orp* significantly inhibited the ability of breast tumors to metastasize to lung tissues.

### Effects of Orp in macrophage polarization in breast tumor tissues

**Figure 3:**
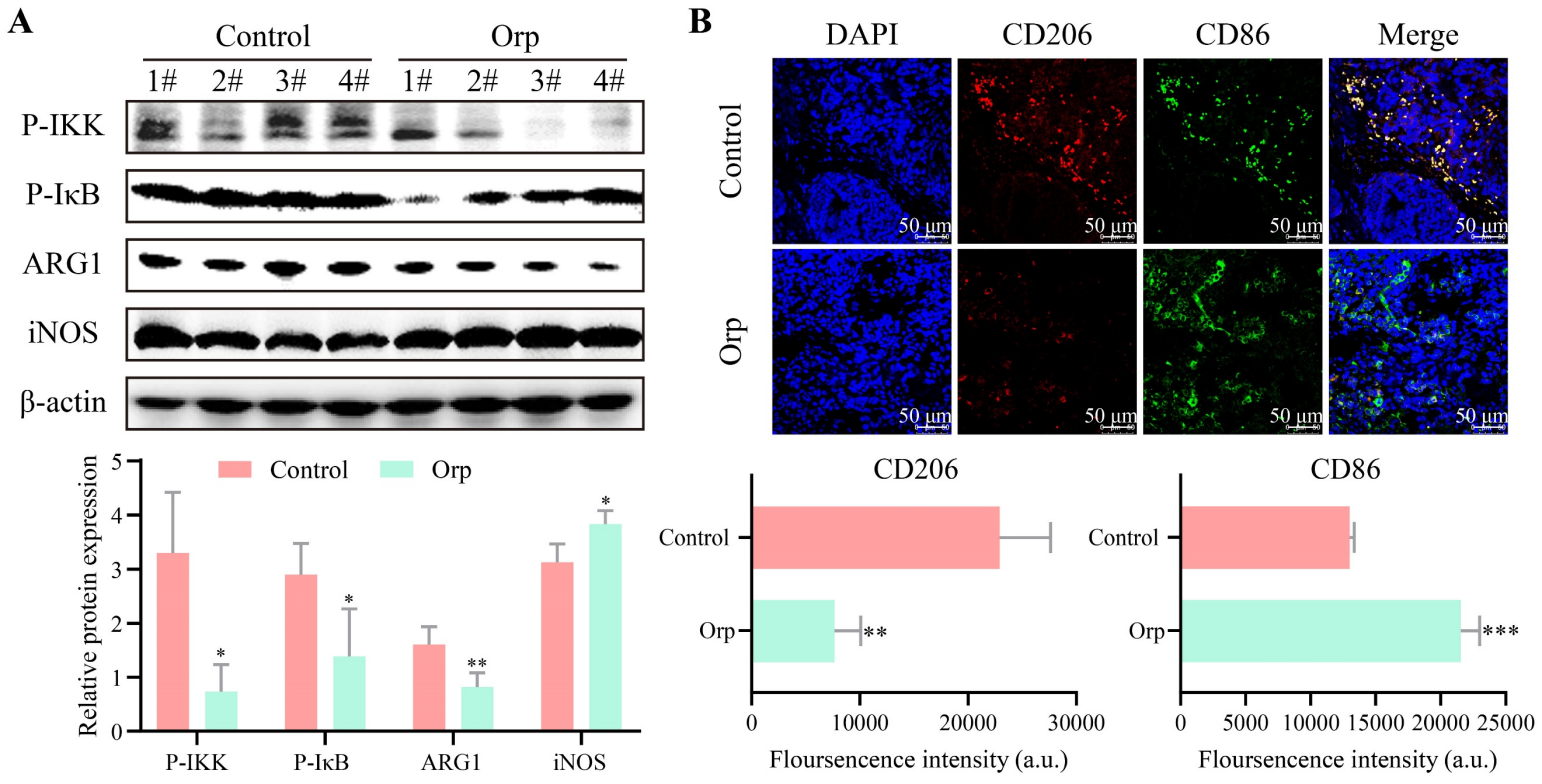
** Alteration of macrophage polarization in the tumor tissues of Orp-treated and control mice. (A)** The expression and semi-quantitative statistics of P-IKK, P-IκB, ARG1 and iNOS proteins in different groups, as monitored by western blotting and ImageJ. The numbers represent the protein obtained from mice in the different groups (n=4).** (B)** Immunofluorescence was used to detect the number of CD206- and CD86-positive cells in the Orp group and control group; The CD206 (red)- and CD86 (green)-positive cells indicate M2 and M1 macrophages, respectively. Data are presented as means ± SD, n≥3. **P<0.01. ***P<0.001.

To investigate whether *Orp*-induced breast tumor cell apoptosis was associated with immunomodulation, the alteration of inflammation signaling in tumor tissues was first measured. As shown in Figure 3A, phosphorylated-IKK and phosphorylated-IκB were expressed at high levels in the control tumor group, but were notably underexpressed in the *Orp*-treated group (Figure 3A, bands 1 and 2). Phosphorylated-IKK and phosphorylated- IκB are well known to be induced upon an imbalance in macrophage polarization. To further confirm the effects of *Orp* on macrophage polarization, western blotting was performed. Arg1 was significantly downregulated in the *Orp*-treated group, whereas iNOS expression was significantly elevated in the *Orp*-treated group (Figure 3A, bands 3 and 4). The data further confirmed that *Orp* could regulate the phenotype of macrophages in the tumor microenvironment and affect their differentiation. Immunofluorescence results also suggested that the number of CD206 (red, labeled M2 macrophage)-positive cells in the control group was significantly higher than that in the *Orp* group, and the number of CD86 (green, labeled M1 macrophage)-positive cells was fewer in the control group. This showed the presence of more CD86-labeled cells in the *Orp*-treated group (Figure 3B). These results confirmed that the *Orp* challenge promoted M1 macrophage polarization and inhibited M2 polarization in breast tumor tissues. Possibly, the *Orp*-induced cytotoxic effects in breast tumors may be related to the *Orp*-mediated repolarization between M1 and M2 macrophages.

### WNT/β-catenin signal transduction in tumor tissues and breast cancer cells

**Figure 4:**
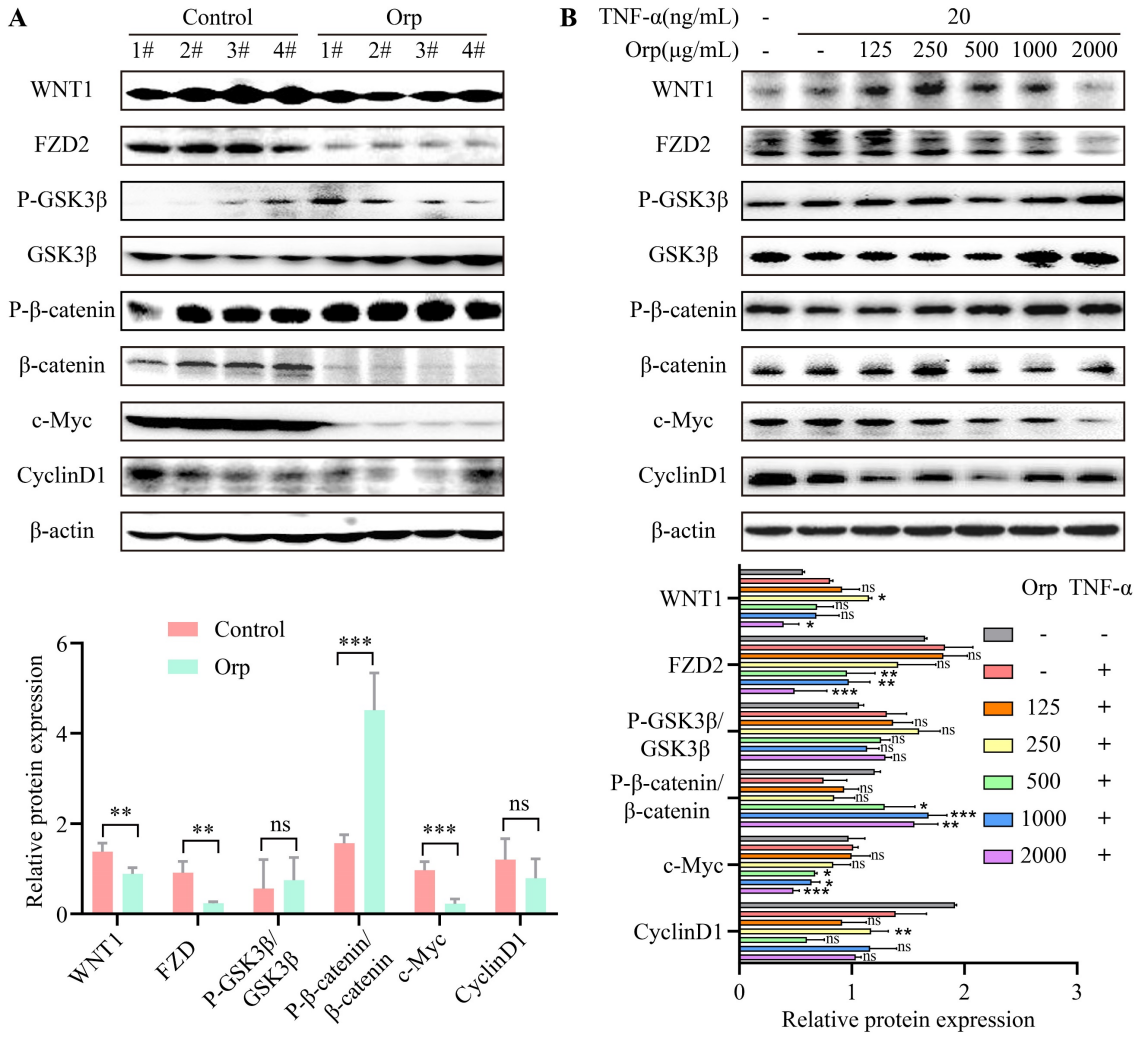
** Orp-mediated changes in WNT/β-catenin signal transduction in tumor tissues and breast cancer cells. (A)** The semi-quantitative protein expression statistics of Wnt1, FZD2, GSK3β, P-GSK3β, β-catenin, P-β-catenin, c-Myc, and cyclin-D1 in the Orp-treated group and control group, as detected by western blotting, in tumor tissues. **(B)** The semi-quantitative protein expression statistics of Wnt1, FZD2, GSK3β, P-GSK3β, β-catenin, P-β-catenin, c-Myc, and cyclin-D1 in breast cancer cells in the Orp plus TNF-α-treated group and control group, as detected by western blotting. Data are presented as means ± SD, n≥3. ns, no significance. *P<0.05. **P<0.01. ***P<0.001.

The WNT/β-catenin signaling pathway is a crucial switch during macrophage polarization in tumor cells. Studies have indicated that the activation of WNT/β-catenin signaling promotes M2 macrophage polarization, which results in tumor growth, migration, metastasis, and immunosuppression in HCC 27. WNT/β-catenin signaling regulates the self-renewal and migration of CSCs, thereby promoting tumor growth and metastasis in breast cancer 21, 28. To verify the mechanism underlying the inhibitory effect of *Orp* breast cancer inhibition, we evaluated the expression of WNT/β-catenin signaling-related proteins in mouse breast cancer tumor tissues and TNF-α-challenged MCF-7 breast cancer cells. Upon comparison, we found no significant difference between the protein expression of GSK3β and cyclin-D1 in the *Orp*-challenged group (Figure 4A, the top band and sixth band). *Orp* treatment significantly inhibited the expression of WNT1, FZD2, β-catenin, and c-Myc (Figure 4A) compared to that in control tumor tissues. Of note, the total β-catenin protein expression was blocked upon treatment with *Orp* compared to that in the control group but exerted no significant effect on the upregulation of phosphorylated β-catenin (Figure 4A, bands 5 and 6). Related reports indicate that the NF-κB and WNT/β-catenin pathways are activated in TNF-α-induced inflammatory responses, leading to the nuclear translocation of p65 and β-catenin as well as the promoter activity of NF-κB and TCF/LEF transcription factors 29. In the *in vitro* experiment, upon treatment with the combination of TNF-α (20 ng/mL/30 min) and *Orp*, MCF7 cells exhibited an obvious inhibition of the concentration gradient of WNT/β-catenin signaling pathway-related proteins, such as WNT1, FZD2, β-catenin, and c-Myc (Figure 4B, bands 1, 2, 6, and 7). This was consistent with the results of animal experiments. The results indicated that the tumor suppressor activity induced by *Orp* in breast cancer is associated with the inactivation of the WNT/β-catenin signaling pathway.

### Role of Orp in the TNF-α-induced activation of WNT/β-catenin signaling

In WNT signaling, ubiquitination is a key mechanism for regulating the quantity of β-catenin. In the absence of WNT protein, β-catenin phosphorylated by CK1 and GSK3 is ubiquitinated by the binding of β-Trcp, an E3 ligase, and subsequently degraded by the 26S proteasome system 30-32. To explore the mechanism through which *Orp* degrades β-catenin, TNF-α was used to construct an inflammatory cell model in MCF-7 cells, and the proteasome inhibitor MG132 was used to treat breast cancer cells under stimulation by TNF-α and *Orp*. Under the effect of TNF-α, MG132 addition activated the β-catenin/c-Myc/cyclin-D1 signaling axis in MCF7 cells (Figure S3A, fourth row vs first row), whereas *Orp* treatment inhibited it. However, this inhibitory effect induced by *Orp* was reversed by MG132 treatment (Figure S3A, third rows VS fifth row). Of note, sub-cellular fractionation also confirmed our observation, as TNF-α treatment promoted the transport of activated β-catenin to the nucleus (Figure S3B, third row vs second row). However, combined treatment with TNF-α and *Orp* did not inhibit the enhancement of nuclear β-catenin expression (Figure S3B, fourth row vs third row), but the degradation of β-catenin in the cytoplasm was significantly inhibited by *Orp* (Figure S3B, eighth row vs seventh row). HSP60 protein served as the internal control for cytoplasmic proteins, and PARP served as the internal control for nuclear proteins (Figure S3B, bands 2 and 3).

### Orp promotes the CRM1-mediated exportation of β-catenin into the cytoplasm, where the β-catenin level is regulated by degradation

**Figure 5:**
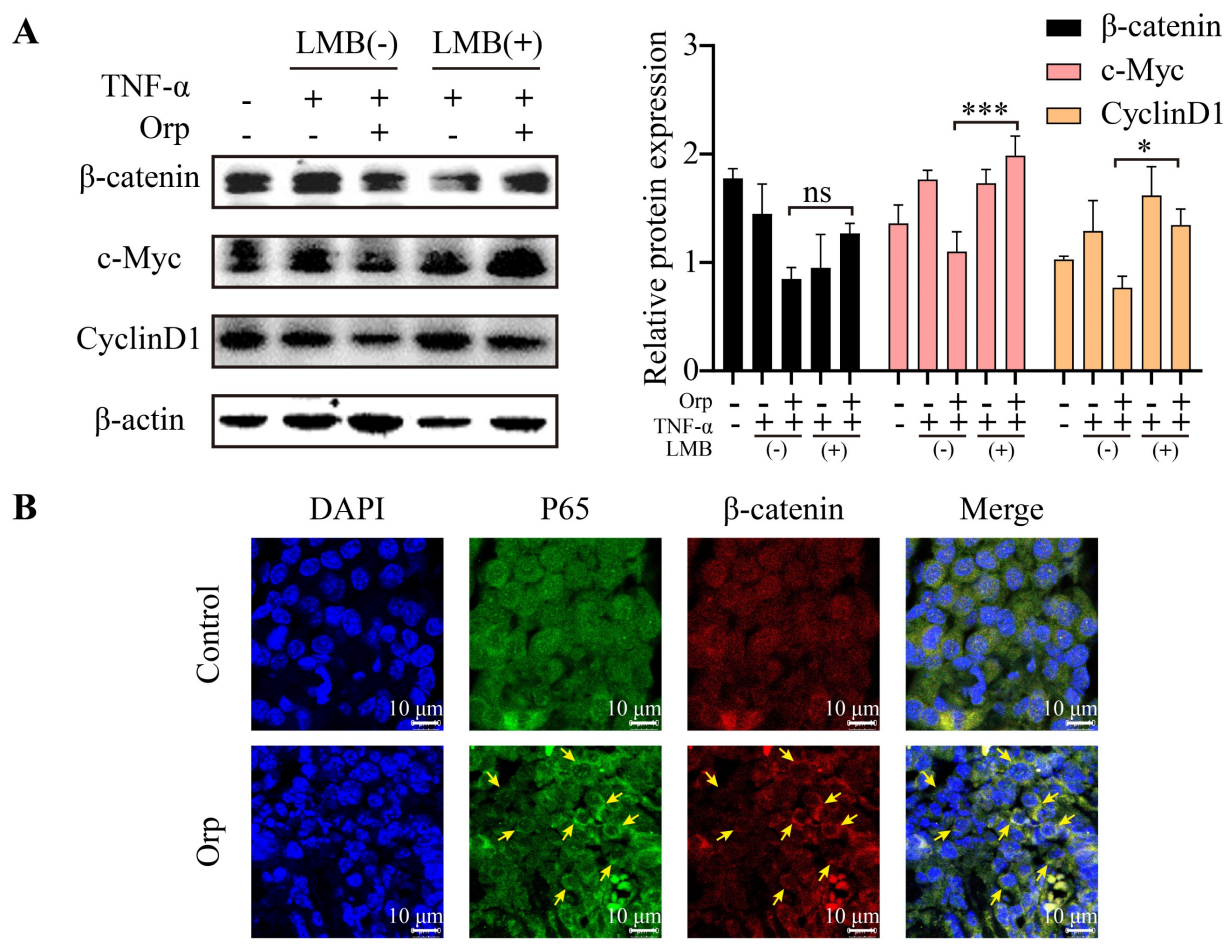
** Orp promotes the CRM1-mediated export of β-catenin into the cytoplasm, where the β-catenin level is regulated by degradation. (A)** The effect of specific CRM1 inhibitor LMB (2.5 ng/mL) on β-catenin expression. MCF-7 cells treated with LMB and Orp in the presence of TNF-α for the indicated duration and analyzed for β-catenin, c-Myc, and cyclin-D1 expression by western blotting and semi-quantitative statistics.** (B)** Immunostaining of β-catenin (red) and P65 (green) proteins in breast tumors from the Orp-treated group and control group. Treatment with a Cy3-conjugated secondary antibody for β-catenin detection or an anti-P65 antibody, followed by treatment with an FITC-conjugated secondary antibody. Nuclei were detected by DAPI (blue) staining. Immunostaining was visualized by confocal microscopy, and the images merged. *P<0.05. ***P<0.001.

Nucleocytoplasmic trafficking pathways in particular are involved in several pathological conditions in cancer, in which the functions of key transporter proteins are counteracted or altered 33.Chromosome Region Maintenance 1 (CRM1), a nuclear transporter protein, mediates the export of approximately 220 proteins and mRNA across the nuclear envelope (NE) and is involved in the regulation of processes associated with cell proliferation, including cell cycle progression and apoptosis 34, 35. CRM1 also is the sole nuclear exporter of several tumor suppressor proteins and growth regulatory proteins, including p53, p21, p73, Rb1, adenomatous polyposis coli (APC), BCR-ABL, FOXO, and STAT3 33, 35, 36. APC regulates the expression of β-catenin, a major component of the WNT signaling pathway, and suppresses tumor progression. In healthy cells, APC chaperones β-catenin and promotes its CRM1-mediated export into the cytoplasm, where the β-catenin level is regulated by degradation 37, 38. Interestingly, when MCF-7 breast cancer cells were treated with leptomycin B 39, 40 (LMB; the first specific inhibitor of CRM1, also known as elactocin, mantuamycin, and NSC 364372, a polyketide isolated from *Streptomyces*) under combined treatment with TNF-α and *Orp* to inhibit β-catenin, *Orp* could not inhibit the expression of β-catenin. This further indicated that the effect of *Orp* on β-catenin occurs in the cytoplasm (Figure 5A, fifth rows vs third row). Further analysis revealed that *Orp* caused the redistribution of nuclear p65 and β-catenin to the cytoplasm, whereas, in the control group animal model, a substantial nuclear accumulation of p65 and β-catenin was observed (Figure 5B). These results suggest that the inflammatory factor-induced activation of WNT/β-catenin and inflammation signaling can be partly blocked by *Orp*. Possibly, *Orp* suppresses the progression of breast cancer by inhibiting the activation and transportation of β-catenin to the nucleus.

## Discussion

**Figure 6:**
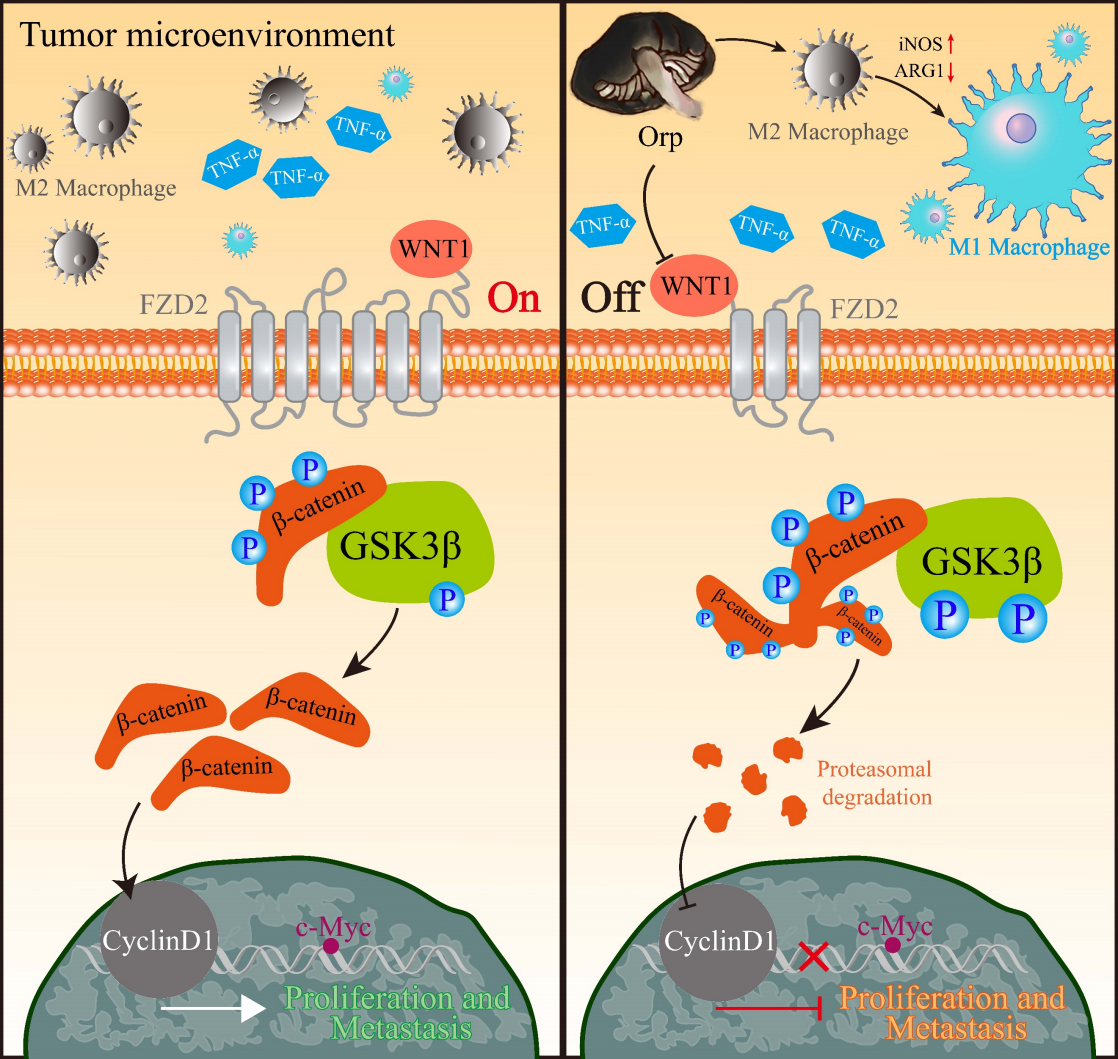
** Representative image of the primary anti-tumor pharmacological mechanism of Orp-derived β-glucans in breast cancer.** In tumor microenvironment, M2 macrophage was enriched in breast tumorigenesis. Besides, TNF-α could prevent β-catenin degradation, which in turn activated the WNT/β-catenin pathway and facilitated nuclear β-catenin accumulation. The nuclear accumulation of β-catenin contributed to the expression of CyclinD1, which subsequently promoted the transcriptional expression of c-Myc, resulting in the promotion in proliferation and metastasis of breast cancer cells. However, Orp administration converted M2 macrophages to M1 macrophages, and also lead to cytosolic β-catenin degradation and the transcriptional inhibition of WNT/β-catenin pathway, which facilitated the blocking signals in c-Myc and the subsequent tumor growth and metastasis.

In this study, we provided several novel insights into the function of *Orp* in breast cancer progression. We found that the extract regulates macrophage polarization, cell proliferation, apoptosis, and metastasis. We first showed that *Orp* boosts the polarization of M2 macrophages to M1 macrophages through NF-κB. Additionally, we observed a positive feedback mechanism involving *Orp* and β-catenin, which aided our understanding of deregulated WNT/β-catenin signaling in the breast cancer microenvironment (Figure 6). More importantly, the regulatory effects of *Orp* in macrophage polarization and the nucleocytoplasmic trafficking of β-catenin have prognostic significance, and these are potential therapeutic targets in novel anti-metastatic treatment for breast cancer.

Glucans belong to a group of polysaccharides localized to the cell wall of bacteria, fungi (including mushrooms), and grains such as barley and oats. Glucans are considered to be biological response modifiers with immunomodulatory and health-boosting effects, including anti-cancer properties 41. In this study, we demonstrated that glucan derived from *Orp* notably inhibited the proliferation and metastasis of breast tumors and promoted the apoptosis of breast tumor cells by regulating macrophage polarization and WNT/β-catenin signal transduction. Our findings provide an important reference for the treatment of breast cancer using products available naturally.

In the present study, glucan extracted from* Orp* inhibited tumor growth in PyMT transgenic breast cancer mice by inducing tumor cell apoptosis and inhibiting tumor cell proliferation. This finding adds to the limited data available on the role of *Orp*-derived polysaccharides in breast tumor treatment. Evidence from related studies has shown that β-glucan can interfere with tumor lung metastasis. For example, in the experimental lung metastasis of colon 26-M3.1 cancer or B16-BL6 melanoma cells, β-glucan derived from yeast significantly inhibited lung metastasis in a dose-dependent manner 42, and β-glucan from *Lentinus edodes* inhibits breast cancer progression and lung metastasis *via* the Nur77/HIF-1α axis 43. We also observed a few inflammatory cells and inflammatory lesions and low expression levels of matrix metalloproteinases in the lung tissues in the *Orp* group, indicating the ability of the extract to inhibit breast tumor metastasis to lung tissues. These results reflect the therapeutic potential of *Orp* in metastatic breast tumors.

Our results indicated that the population of M2 macrophages in the *Orp* group was significantly smaller than that in the control group, whereas the population of M1 macrophages was notably larger in the *Orp* treatment group compared to that in control tumor tissues. Concurrently, Arg1 was significantly downregulated and iNOS expression was significantly elevated in the *Orp*-treated group. Possibly, *Orp* regulates the secretion of inflammatory factors and disrupts the balance in the tumor microenvironment by inhibiting the transformation of M1 macrophages to M2 macrophages, eventually inhibiting the progression and metastasis of breast cancer.

Possibly, in *in vivo* experiments, the administration of *Orp* neutralized the inflammatory microenvironment in tumor tissues, thereby blocking inflammation factor-mediated macrophage polarization and subsequently damaging the WNT/β-catenin signaling cascade. Interestingly, *Orp* induced low levels of total GSK3β and p-GSK3β protein expression both *in vitro* and *in vivo*. Potentially, the elevation of GSK3β activity was owing to the increase in total GSK3β expression. Of note, the degradation of β-catenin is mediated by the phosphokinase activity of GSK3β 44-46. The ratio between GSK3β and p-GSK3β declined in the *Orp*-treated group, indicating the negative association between *Orp* treatment and GSK3β activity. Collectively, this study was the first to confirm the association between *Orp* and WNT/β-catenin signaling pathways in breast cancer and provide a theoretical basis for targeting WNT signaling in the treatment of breast tumors.

In summary, our findings demonstrate the profound antitumor effect of *Orp in vivo*. The extract is capable of “conditioning” an immunosuppressive tumor environment to facilitate antitumor therapeutic effect. However, the *in vivo* antitumor activity and immunostimulatory effect of *Orp* endotoxin-free extract with a high purity and excellent solubility has not been reported previously. The active ingredient in the dextran extract that is responsible for these effects is yet to be identified, and the differences between species are yet to be determined. This makes it necessary for clinical studies on the role of *Orp* extract in cancer therapy. Conversely, questions regarding the precise molecular and cellular events triggered by* Orp* remain. The functions of *Orp* as a ligand are yet to be determined. Thus, additional studies are warranted to further investigate the molecular action of* Orp in vivo* and the feasibility of exploiting this novel immune modulator to disrupt tumor-induced immune tolerance in clinical applications. Our findings suggest that direct macrophage activation by *Orp* may further trigger T cell or DC activation through a signaling pathway that is yet to be defined. Since *Orp* exhibits excellent potential in inducing M1 macrophages, our future investigations will also focus on combining *Orp* treatment with anti-CCL2/CCR2, anti-PD-1, anti-PD-L1 or anti-CTLA-4 checkpoint blockade.

## Conclusion

Collectively,* Orp* treatment inhibited breast tumor growth and restricted the metastasis of breast tumors to lung tissues *in vivo*.* Orp* inhibited the transformation of M1 macrophages into M2 macrophages in tumor tissues and regulated WNT/β-catenin signaling. Our results provide novel insights into the mechanism underlying *Orp* -mediated tumor suppression. Possibly, glucan from *Orp* may be used for treating patients with lung metastases from breast cancer.

## Supplementary Material

Supplementary figures.

## Abbreviations

Orp: Oudemansiella raphanipes
TAMs: tumor-associated macrophages
LNT: Lentinus edodes
ER: estrogen receptor
COX2: cyclooxygenase-2
iNOS: inducible nitric oxide synthase
Arg1: Arginase 1
EMT: epithelial-mesenchymal transition
