# Supplementary material for: Glucan from Oudemansiella raphanipes suppresses breast cancer proliferation and metastasis by regulating macrophage polarization and the WNT/β-catenin signaling pathway
Source: J Cancer. 2024 Jan 1;15(5):1169–81. doi: 10.7150/jca.89873 (PMC10861828; doi:10.7150/jca.89873)
Supplement: Supplementary file 1 — Supplementary figures. [file jcav15p1169s1.pdf]

Supplementary figure legends

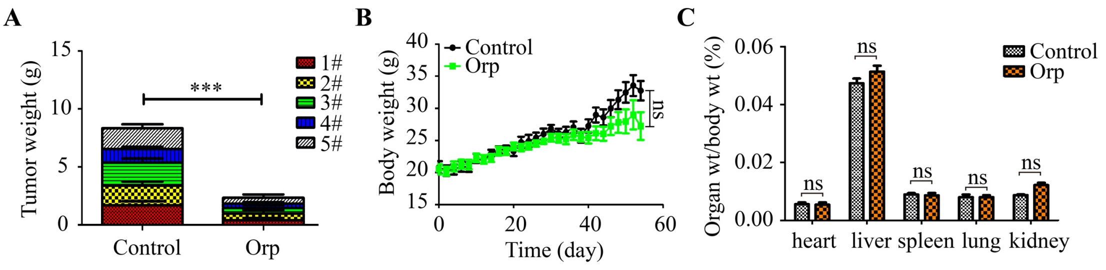

Supplementary Figure 1 Statistical data of the effects of β-glucan derived from Orp on tumor growth and other organs in MMTV-PyMT transgenic breast cancer. (A) Tumor weight statistics for the five paired mammary glands in *Orp*-treated and control group. **(B)** The body weight of mice was measured every 5 days after Orp treatment. Statistical data for body weight of mice in the *Orp*-treated and control groups at different times. **(C)** Organ weight statistics (heart, liver, spleen, lung, and kidney) in the Orp-treated and control groups. Data are presented as means ± SD, n⩾3. $*P < 0.05$.

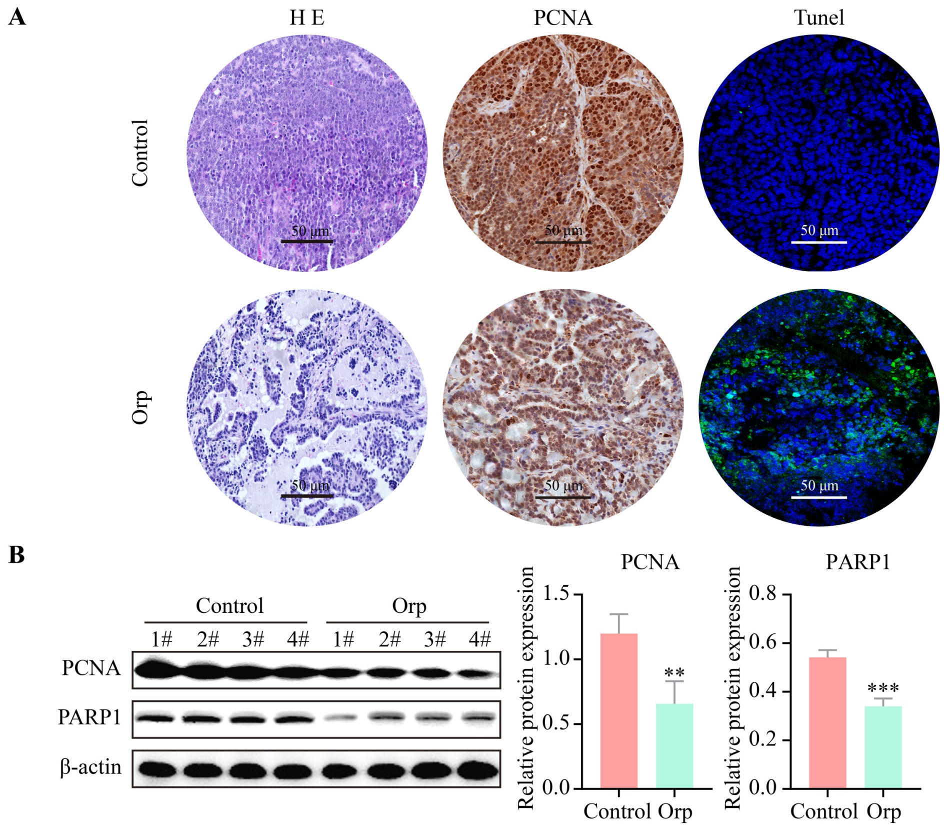

Supplementary Figure 2 Impact of *Orp* on cell proliferation and apoptosis in tumor tissues. (A) HE staining and immunohistochemistry results of mammary tumors were used to detect the expression of proliferation-associated proteins using PCNA in the two groups (Scale bar:

50 μm); TUNEL assay was also used to assess tumor cell apoptosis in the tumor tissues of the two groups (Scale bar: 50 μm). **(B)** The expression of PCNA and PARP1 proteins in different groups, as analyzed by western blotting, and their semi-quantitative statistics (n=4). Data are presented as mean ± SD, n⩾3. ***P<0.001.

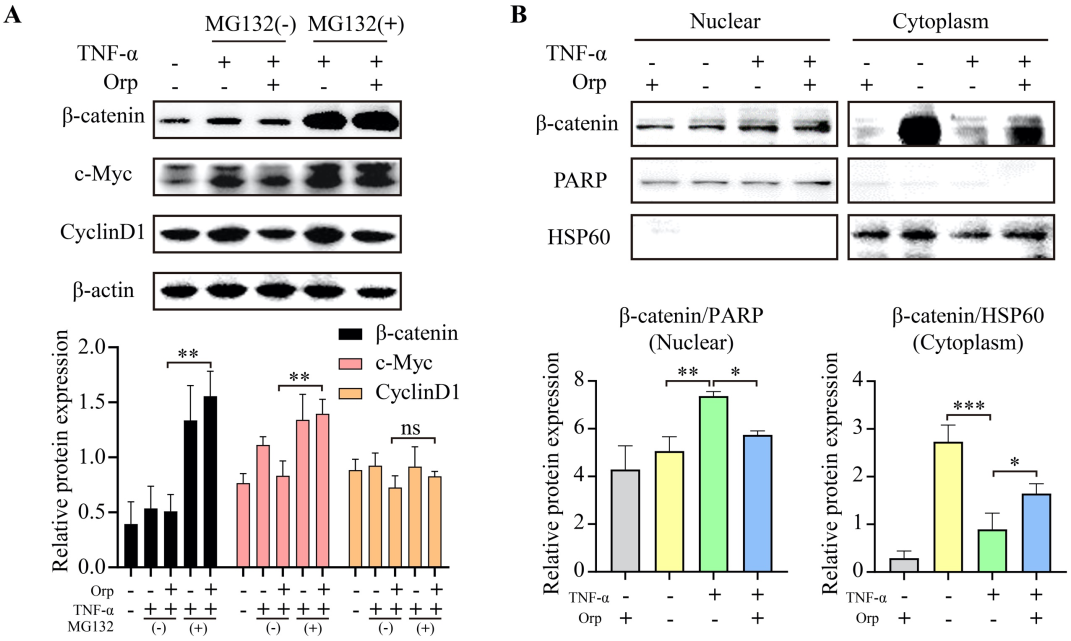

Supplementary Figure 3 Role of the ubiquitin proteasome pathway in the Orp-mediated degradation of β-catenin. (A) The semi-quantitative expression statistics of β-catenin, c-Myc, and cyclin-D1 proteins, as analyzed by western blotting, in MCF-7 cells treated with MG132 and Orp in the presence of TNF-α. Data are shown as means +SD, n⩾3. **(B)** Sub-cellular fractionation followed by immunoblot analysis and semi-quantitative statistics confirmed that β-catenin was enriched in the cytosolic fraction upon treatment with Orp and TNF-α than upon treatment with only TNF-α. PARP and HSP60 are used as nuclear and cytosolic markers, respectively.
